# Synthesis and Immunological Evaluation of Virus-Like Particle-Milbemycin A_3_/A_4_ Conjugates

**DOI:** 10.3390/antibiotics6030018

**Published:** 2017-09-11

**Authors:** Andris Zeltins, Māris Turks, Dace Skrastina, Jevgeņija Lugiņina, Ieva Kalnciema, Ina Balke, Ērika Bizdēna, Vitalijs Skrivelis

**Affiliations:** 1 Latvian Biomedical Research and Study Centre, Ratsupites 1, LV-1067 Riga, Latvia; daceskr@biomed.lu.lv (D.S.); ieva.kalnciema@biomed.lu.lv (I.K.); inab@biomed.lu.lv (I.B.); 2 Institute of Technology of Organic Chemistry, Riga Technical University, P. Valdena Str. 3, LV-1048 Riga, Latvia; maris.turks@rtu.lv (M.T.); jevgenija.luginina@rtu.lv (J.L.); erika.bizdena@rtu.lv (Ē.B.); 3 PharmIdea Ltd., Rupnicu 4, Olaine, Riga District, LV-2114 Olaine, Latvia; pharmidea@pharmidea.lv

**Keywords:** macrocyclic lactone, plant virus, hydrophobic hapten, ELISA

## Abstract

Milbemycins are macrolide antibiotics with a broad spectrum of nematocidal, insecticidal, and acaricidal activity. To obtain milbemycin A_3_/A_4_ derivatives suitable for chemical conjugation to protein carriers (milbemycin haptens), succinate linker and a novel 17-atom-long linker containing a terminal carboxylic acid group were attached to the milbemycin core in a protecting group-free synthesis. The obtained milbemycin A_3_/A_4_ derivatives were coupled to Potato virus Y-like nanoparticles by the activated ester method. The reaction products were characterized and used in mice immunization experiments. It was found that the mice developed weak specific immune responses toward all tested milbemycin haptens.

## 1. Introduction

Milbemycins are a family of 16-membered macrolide antibiotics with a broad spectrum of nematocidal, insecticidal, and acaricidal activity [1]. These structurally similar compounds are produced by various *Streptomyces* strains, which produce, among other derivatives, a mixture of milbemycin A_3_ and A_4_ ([2], and references cited herein). These milbemycins differ in methyl (A_3_) or ethyl (A_4_) substituent at the C-25 atom, and their mixture is directly used as acaricides (Milbemectin) in crop protection. The acaricides also serve as starting materials for semisynthetic production of milbemycin 5-hydroxyimino derivatives, resulting in a commercial product, Milbemycin oxime, suitable for veterinary purposes [3]. Taking into account acceptable pharmacokinetic and safety properties, milbemycin derivatives are also considered as potential drugs in human medicine [4].

Milbemycins elicit persistent paralysis in arthropods and nematodes. This unique mode of action has been exploited to elaborate a screening method for milbemycin-producing *Streptomyces* strains, using survival tests of *C. elegans* [5]. However, the analytical procedures used to test these organisms in vivo, are not easily applicable for high throughput screening.

Alternatively, milbemycins can be quantified by various physical, chemical and chromatographic methods. These include high-performance liquid chromatography with fluorescence detection and liquid chromatography with tandem mass spectrometry detection, as well as thin-layer chromatography [1]. For these approaches, the sample preparation is a critical step: new procedures are still being developed to pre-concentrate the analyte and increase the selectivity [6]. Also, these methods are not suitable for screening of a large number of samples.

Methods of high-throughput analysis are often based on the use of specific antibodies. In the preceding decades, many polyclonal and monoclonal antibodies have been produced in laboratory animals using so-called “hapten technology” to develop enzyme-linked immunosorbent assays (ELISA) for a specific and inexpensive detection of different antibiotics in environmental and food samples [7,8]. For example, moxidectin, a compound structurally similar to milbemycins, can be detected and quantified by a commercial ELISA test in milk, fat and muscle samples [1]. Additionally, monoclonal antibodies were raised against avermectin hapten; the obtained antibodies also recognized ivermectin and other structural analogues [9]. However, to the best of our knowledge, ELISA-type tests are not reported for detection and quantification of milbemycins A_3_ and A_4_.

The hapten technology uses different protein carriers for coupling of low-molecular compounds in order to enhance their immunogenicity; for example, Keyhole limpet haemocyanin, bovine serum albumin (BSA); diphtheria and tetanus toxoids, and artificial, non-infectious analogues of different viruses (virus-like nanoparticles, VLPs) [10]. VLPs are broadly used for chemical coupling of different low-molecular compounds and are highly immunogenic (for reviews, see [11,12]). Previously, we constructed new VLPs, derived from Potato virus Y (PVY-VLPs) and demonstrated a high immune response against foreign peptides displayed on the surface of filamentous PVY-VLPs [13]. As PVY-VLPs contain several surface-exposed Lys-residues, these can also be used for chemical coupling of different ligands.

Considering published data on antibody-based tests for analysis of milbemycin-related compounds, we decided to generate milbemycin A_3_/A_4_-specific antibodies, which could be useful for screening of milbemycin-producing micro-organisms. In the event of its success, the developed methodology can serve as an efficient tool for quantification of milbemycin A_3_/A_4_ content in milbemycin-containing bacterial broth. In turn, this can simplify the search for novel, industrially applicable milbemycin-producing bacterial strains. Additionally, such a methodology can be applied for an inexpensive high-throughput analysis of different environmental and food samples. For this purpose, we designed and synthesized milbemycin derivatives suitable for chemical conjugation using activated ester methodology. Then, we coupled milbemycin haptens to PVY-VLPs and BSA, immunized mice and tested the antibody formation after the immunization experiments.

## 2. Results and Discussion

**Figure 1:**
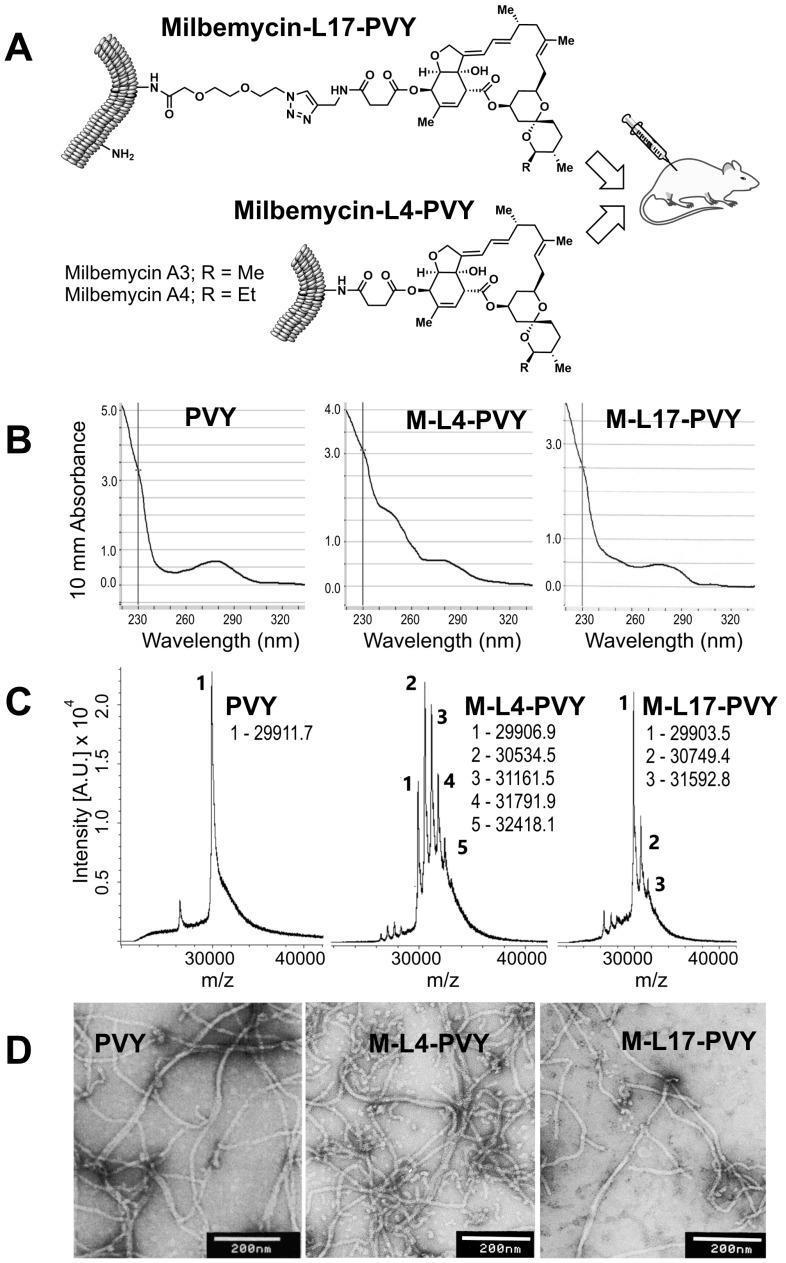
The characterization of synthesized milbemycin-PVY conjugates. (**A**) Chemical structures of milbemycin-L17-PVY (M-L17-PVY) and milbemycin-L4-PVY (M-L4-PVY) used in mice immunization experiments; (**B**) UV spectra of PVY-VLPs (left) and their derivatives M-L4-PVY (in the middle) and M-L17-PVY (right); (**C**) Mass spectrometric analysis of PVY-VLPs (left) and their derivatives M-L4-PVY (in the middle) and M-L17-PVY (right); (**D**) Electron microscopy images of PVY-VLPs (left) and their derivatives M-L4-PVY (in the middle) and M-L17-PVY (right). White bar—200 nm.

To conjugate the milbemycin residues with the amino group containing proteinaceous carriers, it was necessary to introduce the carboxylic acid moiety in the hapten structure. Additionally, the carboxylic acid moiety should be separated from the milbemycin core with a linker that could stimulate the presentation of the hapten on the carrier surface. We synthesized two milbemycin haptens (Figure 1A), using a mixture of naturally occurring milbemycins A_3_ and A_4_ as a starting material [14]. The latter was treated with succinic anhydride, resulting in a selective formation of milbemycin hemisuccinate containing a four atom-long linker (milbemycin-L4, M-L4). The selected experimental conditions were sufficiently mild and the possible formation of known side products such as C(2)-epimer, Δ2,3-isomer [15] or aromatized species [16] was not observed. To create milbemycins with a longer linker, compound M–L4 was amidated with propargylic amine [17] and the resulting product reacted with triethylene glycol-derived azido-acid in copper(I)-catalyzed azide-alkyne cycloaddition reaction. Thus, the protecting group-free approach gave milbemycins A_3_/A_4_ decorated with the 17-atom long linker (milbemycin-L17, M-L17; for the details, see Supplementary Materials File S1).

The solubility of milbemycin derivatives in aqueous solutions is very low (3–4 mg/L). Therefore, the synthesized milbemycin derivatives M-L4 and M-L17, each in a separate experiment, were dissolved in dimethylformamide that reacted with a mixture of solid EDC and sulfo-NHS, as described in Materials and Methods. To find the optimal ratio between M-L4 or M-L17 and PVY-VLPs, increasing amounts of EDC-derivatized milbemycins (approximately 3-, 15- and 75-times molar excess over PVY) were allowed to react with PVY-VLPs. Then, the obtained M-L4-PVY and M-L17-PVY conjugates after dialysis and the clarifications were analyzed by UV spectroscopy, mass spectrometry and electron microscopy (Figure 1).

It was found for both milbemycin derivatives (M-L4-PVY and M-L17-PVY), the excess of 15-times over PVY protein was optimal for efficient coupling, allowing us to obtain the soluble conjugates. On the other hand, the addition of 75-times the excess of milbemycin derivatives in the reaction with PVY resulted in the complete precipitation of VLP material. Additionally, we prepared M-L4 conjugate to bovine serum albumin (BSA) for usage as covering antigen for ELISA tests and to control the antigen for immunizations (Supplementary Materials File S2).

UV spectroscopic analysis of M-L4-PVY and M-L17-PVY conjugates revealed the characteristic increase in the absorption at approximately 245 nm (Figure 1B), which is a known maximum for milbemycin derivatives in their UV spectra [18].

To evaluate the coupling efficiency, mass spectrometric analysis was used to analyze the M-L4-PVY and M17-PVY conjugates (Figure 1C). The identified mass spectral peaks suggested that up to four (M-L4-PVY) or two (M-L17-PVY) milbemycin residues were conjugated to each PVY protein, respectively. If calculated from the peak areas of the mass spectra, M-L4-PVY preparation consists of 92% of the hapten-substituted PVY protein, but the M17-PVY preparation was characterized as consisting of 50% of the conjugated carrier protein.

Considering the importance of the VLP structure for the immune response, we analyzed the samples under electron microscope, as seen from photographs (Figure 1D), PVY-VLPs retain characteristic filamentous VLP structures after chemical modification with M-L4 and M-L17.

For evaluation of immunological activity, BALB/c mice were immunized with high doses of milbemycin-PVY conjugates (50 µg/mouse), as described in Materials and Methods. The results are summarized in Table 1.

The mice immunization experiments resulted in the following conclusions: (1) BSA as a carrier is a weak immunogenic, while PVY-VLP carrier elicits a strong immune response in mice (dilution 1:84,900); (2) chemical coupling leads to the reduction of the immune response against the carrier (antibody titers were reduced up to 10–15 times to 1:5400–7200); conjugated milbemycin molecules possibly mask the natural epitopes of the carrier; (3) milbemycins appear to be weak immunogens, as found after the testing of sera against different covering antigens. The obtained polyclonal antibodies were also tested in dot-blots, and the results confirmed the low or absent reactivity against milbemycin-containing antigens (Supplementary Materials File S3).

The outcome of our designed and synthesized milbemycin haptens was weak immunogens. After immunization with protein–hapten conjugates, a large portion of the produced antibodies appeared to be specific for the conjugate and carrier protein, and only a small part of them recognized the target substance. Additionally, the coupled hydrophobic milbemycins may interact with hydrophobic domains of the carrier protein, resulting in a loss of surface-location of the target antigen, which is necessary for the activation of immune cells [19]. In the case of milbemycin–PVY derivatives, we did not observe VLP disassembly after chemical coupling, suggesting that the overall structure of PVY protein has not changed and milbemycins should be located on the VLP surface. However, milbemycin residues can be internalized within hydrophobic pockets in the particle without influencing the overall structure of the VLP carrier. Therefore, in the future, we plan to test other protein carriers, including different VLPs as coupling partners for milbemycin haptens that are already synthesized. After obtaining higher antibody titers, the monoclonal antibodies against milbemycins A_3_/A_4_ will be generated. Alternatively, production of single chain variable fragment (scFv) antibodies from phage display libraries [20] can be used to generate specific antibodies against milbemycins.

## 3. Materials and Methods

### 3.1. Chemical Synthesis of Milbemycin Derivatives

#### 3.1.1. Synthesis of Milbemycin Hemisuccinate M-L4

DIPEA (33 μL, 0.190 mmol) and DMAP (2 mg, 0.015 mmol) were added to a mixture of milbemycins A_3_/A_4_ (A_3_/A_4_ ~ 40:60) (50 mg, 0.092 mmol) and succinic anhydride (20 mg, 0.202 mmol) in anhydrous DCM (2 mL). The resulting reaction mixture was stirred at ambient temperature and its progress was controlled by HPLC. The solvent was evaporated under reduced pressure and the resulting mixture was chromatographed on silica gel. Product M-L4 was obtained as a colorless oil (55 mg, 93%) with A_3_/A_4_ ratio ~ 1/2. For additional experimental details and product characterization, see Supplementary Materials File S1.

#### 3.1.2. Synthesis of Milbemycin Derivative M–L17

Propargylamine (7 μL, 0.115 mmol) was added at 0 °C to a mixture of milbemycin hemisuccinate M-L4 (67 mg, 0.104 mmol), Et_3_N (38 μL, 0.276 mmol) and CMPI (32 mg, 0.125 mmol) in anh. DCM (4 mL). The resulting reaction mixture was stirred at ambient temperature and its progress was controlled by HPLC. The reaction mixture was washed in 10% aqueous citric acid solution (3 × 1 mL), dried over anh. Na_2_SO_4_, was filtered and then evaporated under reduced pressure. The resulting residue was chromatographed on silica gel and the isolated intermediate propargyl amide (42 mg, 73%; mixture of milbemycins A_3_/A_4_ ~ 1/2) was used in the next step.

A solution of CuSO_4_·5H_2_O (1 mg, 0.004 mmol) in H_2_O (0.5 mL) and a solution of sodium ascorbate (2 mg, 0.008 mmol) in H_2_O (0.5 mL) were sequentially added to a mixture of the above mentioned intermediate propagyl amide (30 mg, 0.044 mmol) and [2-(2-azidoethoxy)ethoxy]acetic acid cyclohexylammonium salt (14 mg, 0.048 mmol) in THF (1 mL). The resulting reaction mixture was stirred at ambient temperature and its progress was controlled by HPLC. Then, DCM (10 mL) was added to the reaction mixture and the resulting mixture was washed with brine (3 × 1 mL), and dried over anhydrous. Na_2_SO_4_, filtered and evaporated under reduced pressure. The residue was chromatographed on silica gel and yielded the expected product, M-L17 as an oily material. The latter was dissolved in freshly distilled dioxane and lyophilized for 8 h at 0.01 Torr to yield the product M-L17 (42 mg, 73%) in a form of a white amorphous foamy material [mixture of milbemycins A3/A4 ~ 1/2].

Reaction schemes, details of the synthesis and analytical data are summarized in Supplementary Materials File S1.

### 3.2. Coupling of Milbemycin Derivatives to Protein Carriers

Milbemycin derivatives M-L4 (3 mg) and M-L17 (3 mg) were separately dissolved in dimethylformamide (DMF; 100 µL) and added to a mixture of solid EDC (1-ethyl-3-[3-dimethyl-aminopropyl] carbodiimide; 2.3 mg) and sulfo-NHS (*N*-hydroxysulfosuccinimide; 2.6 mg). The resulting reaction mixtures were shaken for 4 h at room temperature. Then the excess EDC was neutralized with mercaptoethanol (1.4 µL).

The PVY-VLPs were purified as described previously [13]. To optimize the reaction conditions for M-L4 or M-L17 and PVY-VLPs, the milbemycin-containing reaction mixtures from the previous step (2, 10, and 50 µL) were slowly added to a solution of PVY-VLP (2 mL; 0.5 mg/mL in 100 mM potassium phosphate, pH 7.6) and the resulting mixtures were incubated for 16 h at 4 °C on a rotator mixer. The reactions were stopped by the addition of Tris·HCl (1M, pH 7.0; 20 µL). Then, the obtained M-L4-PVY and M-L17-PVY conjugates were dialyzed against 200 volumes of 1× PBS buffer at 4 °C for 16 h, clarified by centrifugation (16,000 *g*, 10 min), and used for UV spectroscopy, mass spectrometry, electron microscopy analysis and mice immunization experiments.

To obtain the covering antigen for ELISA tests and control the antigen for immunizations, M-L4 conjugate to BSA was prepared in similar conditions (details in Supplementary Materials File S2).

### 3.3. Characterization of Milbemycin Conjugates

The UV spectra of VLP or BSA derivative solutions (0.5 mg/mL) were recorded using a Nanodrop ND-1000 spectrophotometer (NanoDrop Technologies, Wilmington, DE, USA).

For mass spectrometric analysis, the VLP or BSA solutions (0.5 mg/mL) were diluted with a 3-hydroxypicolinic acid matrix solution and were spotted onto an MTP AnchorChip 400/384TF. Matrix-assisted laser desorption/ionization analysis was carried out on an Autoflex MS (Bruker Daltonik, Bremen, Germany).

To obtain electron microscopy images, VLP samples (0.5 mg/mL) were adsorbed on carbon formvar-coated copper grids and were negatively stained with 1% uranyl acetate aqueous solution. The grids were examined using a JEM-1230 electron microscope (JEOL, Tokyo, Japan) at 80 kV.

A BCA Protein assay kit (Thermo Scientific, Rockford, IL, USA) was used for determination of protein concentration.

### 3.4. Immunization

The mice experiments were approved by the Latvian Animal Protection Ethics Committee and the Latvian Food and Veterinary service, permission No. 61/12.05.2014. BALB/c. The mice (groups of 5 animals) were immunized with milbemycin conjugates (50 µg/mouse), mixed with Freund’s complete for 1st or incomplete adjuvants for 2nd and 3rd injection at days 1, 14 and 28. Blood serums were collected at day 42 and analyzed by ELISA tests as described in [13].

## 4. Conclusions

In this work, we have demonstrated a protecting group-free synthesis of a novel milbemycin A3/A4 derivative with a 17-atom long linker for subsequent coupling to proteinaceous carriers. Therefore, the synthesized milbemycin derivatives can be conjugated to plant virus PVY-VLPs with high efficiency, and the resulting milbemycin-VLP conjugates retain the typical viral morphology of PVY-VLPs. However, as mice develop relatively low immune responses against the tested milbemycin haptens, additional experiments are necessary to find the appropriate carrier for milbemycin derivatives to obtain specific antibodies against milbemycins.

## Figures and Tables

**Table 1:** Immune responses against different milbemycin conjugates after mice immunization.

Covering Antigens Used in ELISA Tests	Antigens Used for Immunizations			
	M-L4-BSA	M-L4-PVY	M-L17-PVY	PVY
BSA	85	-	-	-
PVY	-	5400	7200	84,900 *
M-L4-PVY	-	23,000	-	-
M-L17-PVY	-	-	40,000	-
M-L4-BSA	1425	380	160	-
Milbemycin **	140	80	190	-

* Published data [13]; ** unmodified milbemycin A_3_/A_4_ from stock in dimethylformamide (DMF) (10 mg/mL) was diluted in PBS to 0.02 mg/mL and used as covering antigen for microtiter plates; - not tested. The first column contains a list of tested antigens, which were used to test obtained polyclonal antibodies. ELISA plates were covered with corresponding antigens on the microtiter plates (2 µg/well). The numbers in Table 1 show the mean values of endpoint titers, which were defined as the highest serum dilutions, resulting in absorbance values that were three times greater than that of sera obtained from the naïve mice.

## Supplementary Materials

The following files are available online at www.mdpi.com/2079-6382/6/3/18/s1. Electronic Supplementary Material, File S1: Part I. Synthesis of linker-modified milbemycins A3/A4; File S2: Part II. Synthesis of milbemycin-M-L4-BSA conjugate; File S3: Part III. Dot-blot tests.
