# Supplementary material for: Synthesis and Immunological Evaluation of Virus-Like Particle-Milbemycin A3/A4 Conjugates
Source: Antibiotics (Basel). 2017 Sep 11;6(3):18. doi: 10.3390/antibiotics6030018 (PMC5617982; doi:10.3390/antibiotics6030018)

ELECTRONIC SUPPLEMENTARY MATERIAL

Synthesis and immunological evaluation of virus-like particle-milbemycin A_3/A_4 conjugates

Andris Zeltins • Māris Turks • Dace Skrastina • Jevgeņija Lugiņina • Ērika Bizdēna • Ieva Kalnciema • Ina Balke • Vitalijs Skrivelis

Part I. Synthesis of linker-modified milbemycins A_3/A_4

The HO-group at C(5) of milbemycins is practically the only possibility to attach the required linker. On the other hand, we have chosen a linker with carboxylic acid end-group which will be later condensed with the virus-like particles:

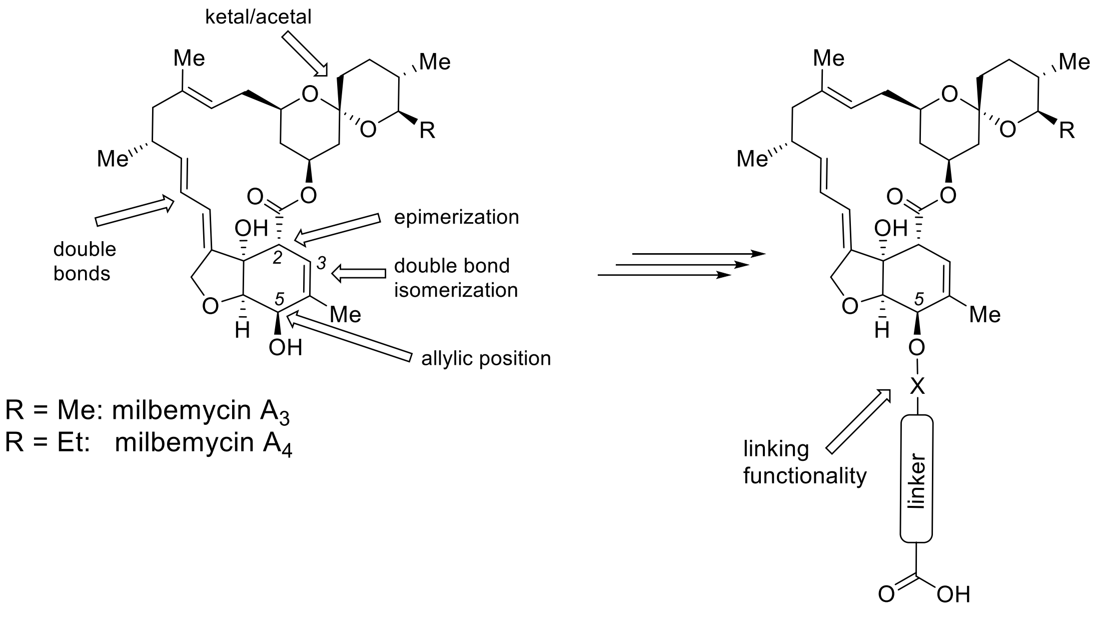

The linker design includes many limitations:

1) Protecting groups (e.g.: benzyl-, Cbz-groups) cleaved by hydrogenation conditions cannot be used due to the presence of double bonds;
2) Protecting groups cleaved under strongly acidic conditions (e.g. TFA) cannot be used due to the presence of acetal moiety and potential elimination of quaternary alcohol which can further lead to aromatization;
3) Protecting groups cleaved under basic conditions cannot be used due to known epimerization at C(2). Additionally, basic conditions induce Δ2,3-

isomerization of double bond in the oxahydrindene portion.[1] Enhanced leaving group ability of functional group attached to C(5) can further lead to aromatization of the cyclohexene ring under basic conditions.[2]

With this information in hand we proceeded to the design of the linker. It appeared that alkylation of C(5)-OH under various conditions (e.g.: propargyl bromide / K_2CO_3) resulted in degradation of starting material. It was proved that basic conditions result in various unwanted transformations with one of them being water elimination and diene formation between C(5) and carboxylic acid moiety followed by aromatization. As next we proceeded to the esterification of C(5)-OH. Approaches with most of condensation agents were not successful. Finally, we have discovered that milbemycin reacts well with succinic anhydride in the presence of DMAP. This process resulted in milbemycin hemisuccinate (**M-L4**) in 93% isolated yield:

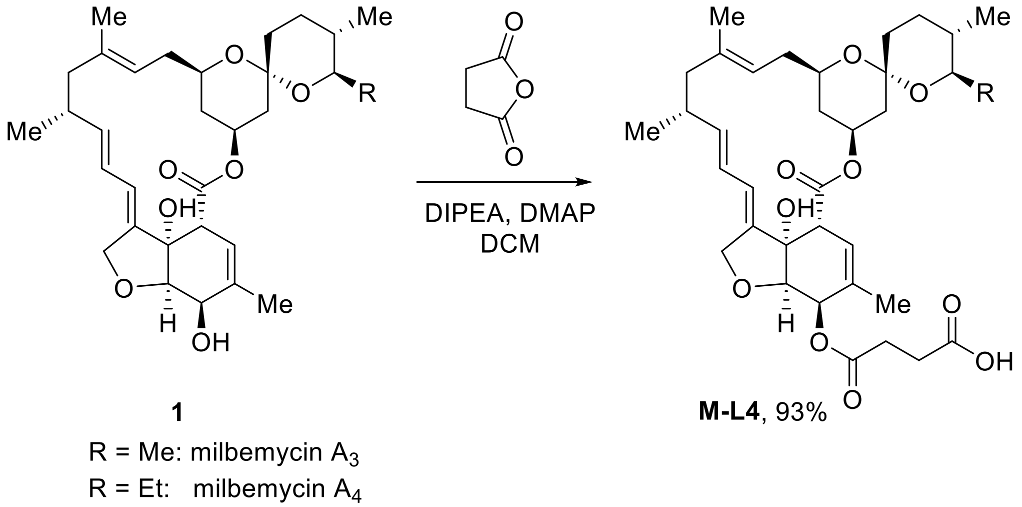

1
R = Me: milbemycin A_3
R = Et: milbemycin A_4

M-L4, 93%

---

[1] a) Awasthi, A.; Razzak, M.; Al-Kassas, R.; Greenwood, D.R.; Harvey, J.; Garg, S. Isolation and characterization of degradation products of moxidectin using LC, LTQ FT-MS, H/D exchange and NMR. *Anal. Bioanal. Chem.* **2012**, *404*, 2203-2222; b) Awasthi, A.; Razzak, M.; Al-Kassas, R.; Greenwood. D.R.; Harvey, J.; Garg, S. Separation and identification of degradation products in eprinomectin formulation using LC, LTQ FT-MS, H/D exchange, and NMR. *J. Pharm. Biomed. Anal.* **2012**, *63*, 62–73; c) Pivnichny, J.V.; Arisen, B.H.; Preiser, F.A.; Shim, J.K.; Mrozik, H. Base-catalyzed isomerization of avermectins. *J. Agr. Food. Chem.* **1988**, *36*, 826–828; d) Moore, A.F.; Lacey, H.J.; Crombie, A.; Lacey, E.; Piggott, A.M. Primary pH degradation products of doramectin. *Tetrahedron Lett.* **2016**, *57*, 4224-4227.

[2] a) Smith III, A.B.; Thompson, A.S. Avermectin-milbemycin studies. 2. An efficient chemical degradation of avermectin B_{1a}. *Tetrahedron Lett.* **1985**, *26*, 4279-4282; b) Mrozik, H.; Eskola, P.; Fisher, M.H.; Egerton, J.R.; Cifelli, S.; Ostlind, D.A. Avermectin Acyl Derivatives with Anthelmintic Activity. *J. Med. Chem.* **1982**, *25*, 658-663.

Compound **M-L4** was chosen as milbemycin containing short linker. However, succinic hemi-ester can be too short to be used in biological chemistry during the building of haptenic structures. For the chain elongation we have chosen the copper(I) catalyzed azide-alkyne cycloaddition reaction (CuAAC). Therefore, we had to install either alkyne or azide "sticky end" into the structure:

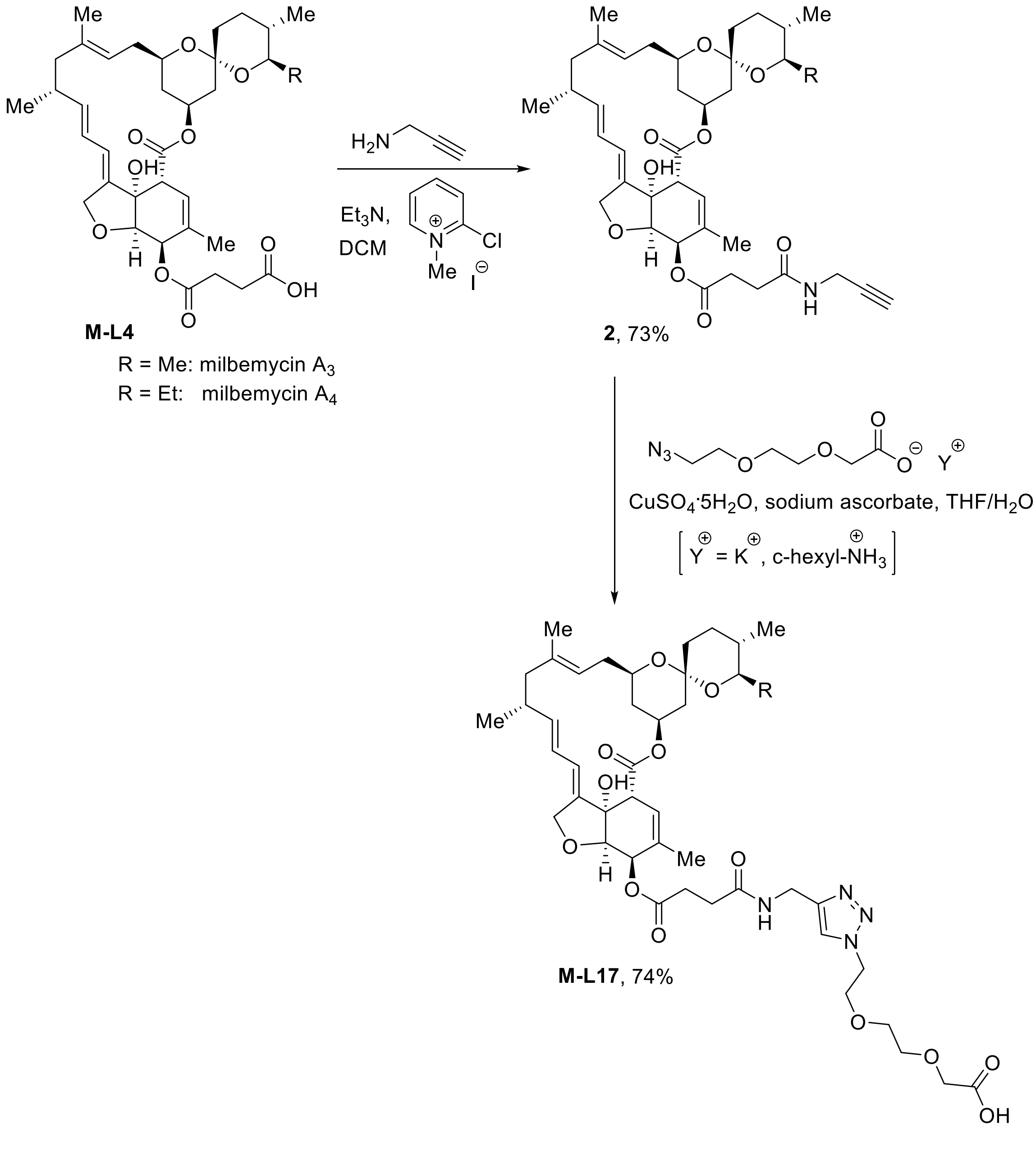

After several attempts we have found that amidation of the carboxylic acid moiety in intermediate **M-L4** proceeds well with propargylamine in the presence of condensation agent 2-chloro-1-methylpyridinium iodide (CMPI). In this way amide **3** was obtained in 73% isolated yield.

Next, we proceeded to elongate the linker *via* CuAAC reaction which works well also in the presence of unprotected carboxylic acid groups or their corresponding salts. In this final step we have used commercially available (company BAPEKS, Latvia) potassium or cyclohexylammonium salt of PEG-derived ω-azido-carboxylic acid ([2-(2-azidoethoxy)ethoxy]acetic acid). The triazole synthesis (74%) finalized the stepwise building of 17-atom-linker-derived milbemycins A_3/A_4 (compound **M-L17**) which now are ready for conjugation with selected biological objects. The developed synthesis does not use any protecting groups.

Experimental part

Synthesis of hemisuccinate M-L4 (process 1 → M-L4)

DIPEA (33 µL, 0.190 mmol) and DMAP (2 mg, 0.015 mmol) were added to a mixture of milbemycins A_3/A_4 (A_3/A_4 ~ 40:60) (0.05 g, 092 mmol) and succinic anhydride (20 mg, 0.202 mmol) in anh. DCM (2 mL). The resulting reaction mixture was stirred at ambient temperature and its progress was controlled by HPLC (t_R(**1**$_{A3}$)=12.210 min, t_R(**1**$_{A4}$)=12.730 min, t_R(**M-L4**$_{A3}$)=11.952 min, t_R(**M-L4**$_{A4}$)=12.424 min).[3] The solvent was evaporated under reduced pressure and the resulting mixture was chromatographed on silica gel (0.5% $CHCl_3$/MeOH). Product **M-L4** was obtained as colorless oil (55 mg, 93%) with A_3/A_4 ratio ~1/2.

Analytical data for compound M-L4 [a mixture of A_3/A_4 ~ 1/2]

^{1}H NMR ($CDCl_3$, 300 MHz): δ = 5.81 - 5.65 (m, 2H), 5.56 - 5.61 (m, 2H), 5.41 - 5.28 (m, 2H), 4.96 (t, *J* = 7.5 Hz, 1H), 4.61 (dd, AB syst., *J* = 1.7, 14.6 Hz, 2H), 4.04 (d, *J* = 5.8 Hz, 1H), 3.61 – 3.51 (m, 1H), 3.36 – 3.30 (m, 1H), 3.10 – 3.03 (m, 1H), 2.79 – 2.60 (m, 4H), 2.49 – 2.35 (m, 1H), 2.28 – 2.13 (m, 3H), 2.09 – 2.00 (m, 1H), 1.90 – 1.46 (m, 13H), 1.40 – 1.23 (m, 4H), 1.03 – 0.81 (m, 11H).

^{13}C NMR ($CDCl_3$, 75.5 MHz): δ = 176.9, 173.3, 171.8, 142.8, 138.9, 137.0, 133.2, 123.4, 120.9, 120.8, 120.2, 97.4, 80.3, 77.3, 76.0, 70.4, 68.8, 68.3, 67.4, 48.5, 45.7, 41.3, 36.5, 35.9, 35.6, 34.6, 34.2, 28.9, 28.7, 27.8, 25.6, 22.3, 19.4, 17.7, 15.5, 10.1.

LC-MS: Calcd for $[C_{36}H_{50}O_{10}+H]^+$ 643.3 [milb. A_4]; found: 643.3

HRMS: Calcd for $[C_{36}H_{50}O_{10} + Na^+]$ 665.3302; found 665.3296 (0.9 ppm).

---

[3] ***HPLC method:***
Sample: reaction mixture (30 µL) in 2 mL MeOH;
Column: Waters XBrige C18 3.5 µm, 4.6 mm × 150 mm;
Column temperature: 40 °C ;
Mobile phase: A: MeCN, B: 0.01 M KH_2PO_4 + 6% MeCN;
Gradient: 10 → 95% A in 10 min, 95% A until 17 min;
Injection volume: 10 µL;
Detection: DAD at 240 nm.

^{1}H-NMR spectrum (300 MHz, $CDCl_3$) of compound M-L4:

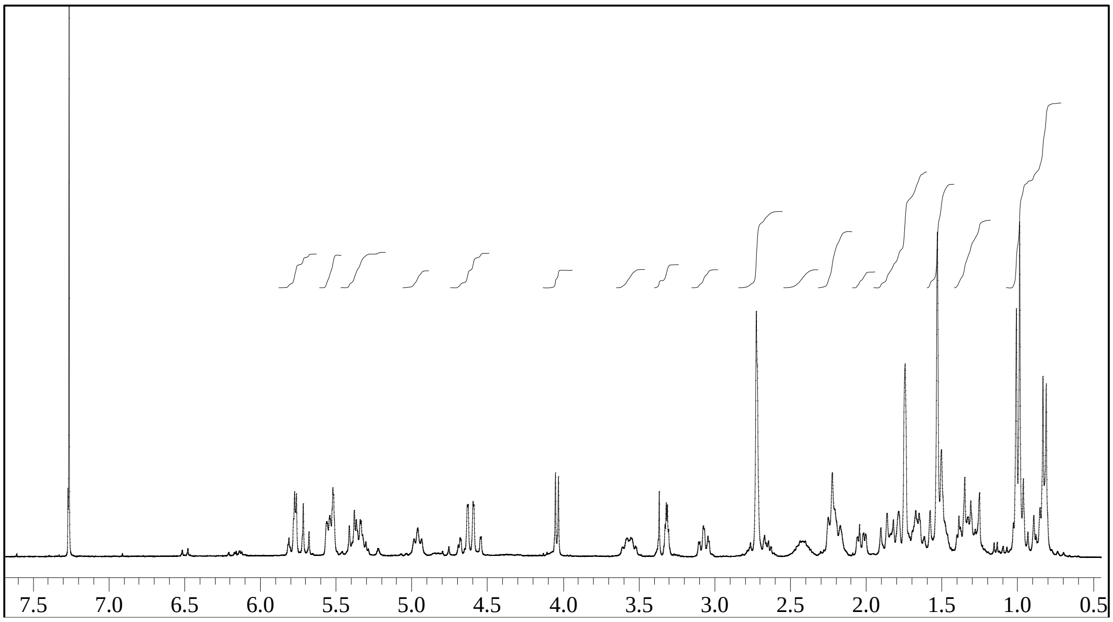

^{13}C-NMR spectrum (75.5 MHz, $CDCl_3$) of compound M-L4:

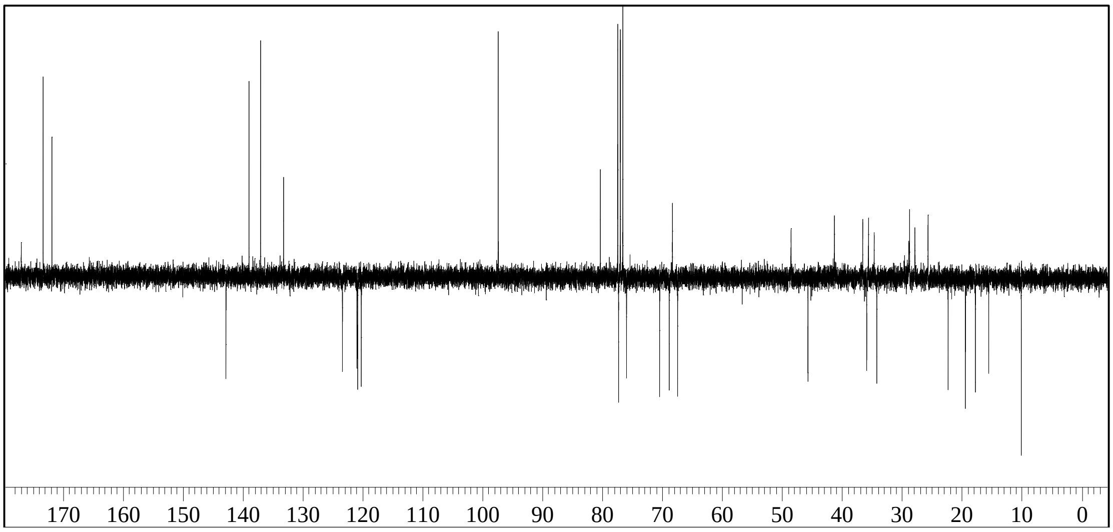

Mass-spectrum of compound M-L4 (LC-MS of milbemycin A_4)

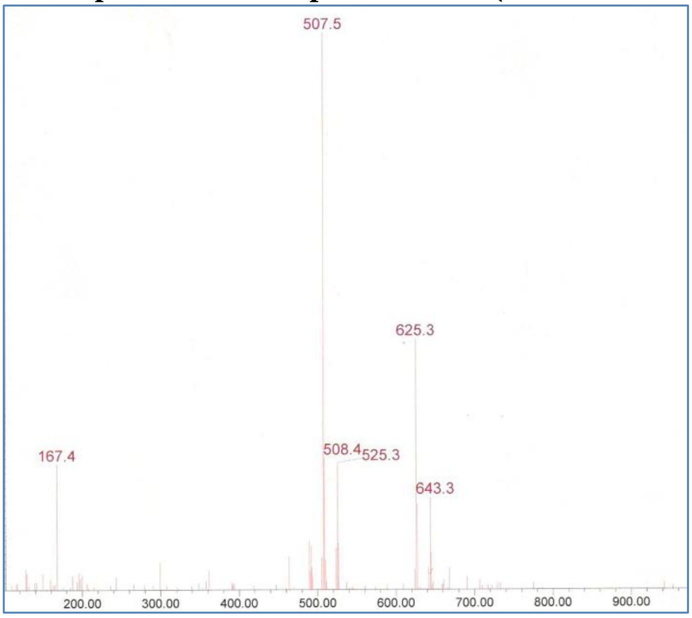

Synthesis of intermediate 2 (process M-L4 → 2)

Propargylamine (7 µL, 0.115 mmol) was added at 0 °C to a mixture of hemisuccinate **M-L4** (67 mg, 0.104 mmol), Et_3N (38 µL, 0.276 mmol) and CMPI (32 mg, 0.125 mmol) in anh. DCM (4 mL). The resulting reaction mixture was stirred at ambient temperature and its progress was controlled by HPLC (for the conditions, see above; t_R(**2**$_{A3}$)=12.116 min, t_R(**2**$_{A4}$)=12.226 min). The reaction mixture was washed by 10% aqueous citric acid solution (3×1 mL), dried over anh. Na_2SO_4, filtered and evaporated under reduced pressure. The residue was chromatographed on silica gel (0.5% $CHCl_3$/MeOH) and yielded product **2** (42 mg, 73%) in a form of colorless oil [mixture of milbemycins A_3/A_4 ~ 1/2].

Analytical data for compound 2 [a mixture of A_3/A_4 ~ 1/2]

^{1}H NMR ($CDCl_3$, 300 MHz): δ = 6.21 – 6.03 (m, 1H), 5.85 – 5.62 (m, 2H), 5.60 – 5.45 (m, 2H), 5.45 – 5.26 (m, 2H), 4.95 (t, *J* = 7.5 Hz, 1H), 4.62 (dd, AB syst., *J* = 2.0, 14.5 Hz, 2H), 4.14 – 3.92 (m, 4H), 3.65 – 3.45 (m, 1H), 3.37 – 3.18 (m, 1H), 3.14 – 2.99 (m, 1H), 2.85 – 2.70 (m, 2H), 2.64 – 2.34 (m, 3H), 2.31 – 2.10 (m, 3H), 2.08 – 1.06 (m, 18H), 1.06 – 0.73 (m, 9H).

^{13}C NMR ($CDCl_3$, 75 MHz): δ = 173.2, 172.3, 171.0, 142.9, 138.8, 136.9, 133.1, 123.27, 121.0, 120.8, 120.2, 97.3, 80.3, 77.3, 75.9, 71.5, 70.3, 68.7, 68.3, 67.4, 48.5, 45.6, 41.3, 36.6, 35.9, 35.6, 34.6, 34.2, 31.1, 29.6, 29.2, 27.8, 25.6, 22.3, 19.5, 19.3, 17.7, 15.5, 10.1.

HRMS (ESI): Calcd for milb.A_3 [$C_{38}H_{51}NO_9$+H]$^+$ 666.3637; found: 666.3635

HRMS (ESI): Calcd for milb.A_3 [$C_{39}H_{53}NO_9$+Na]$^+$ 702.3613; found: 702.3605

^{1}H-NMR spectrum (300 MHz, $CDCl_3$) of compound 2:

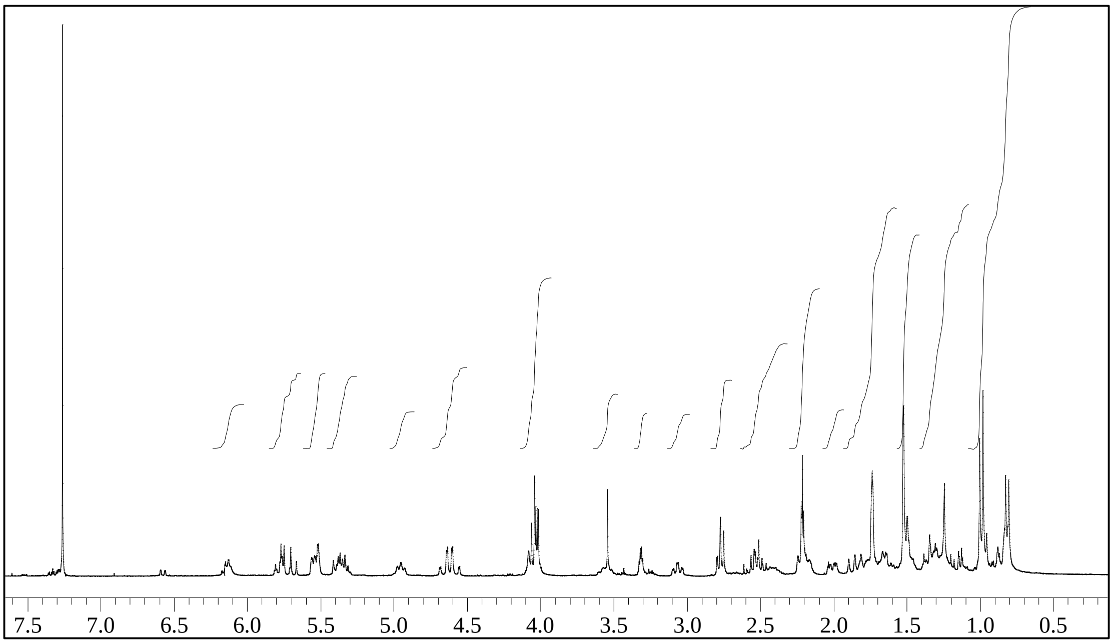

^{13}C-NMR spectrum (75.5 MHz, $CDCl_3$) of compound 2:

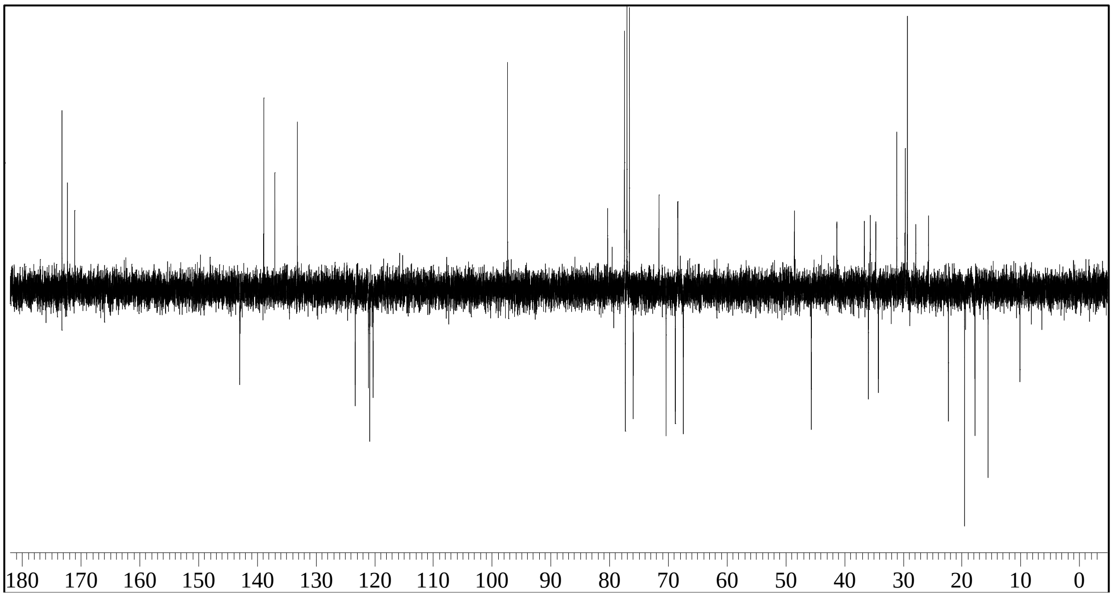

Mass-spectrum of compound 2 $[M+H]^+$ (A_3 form):

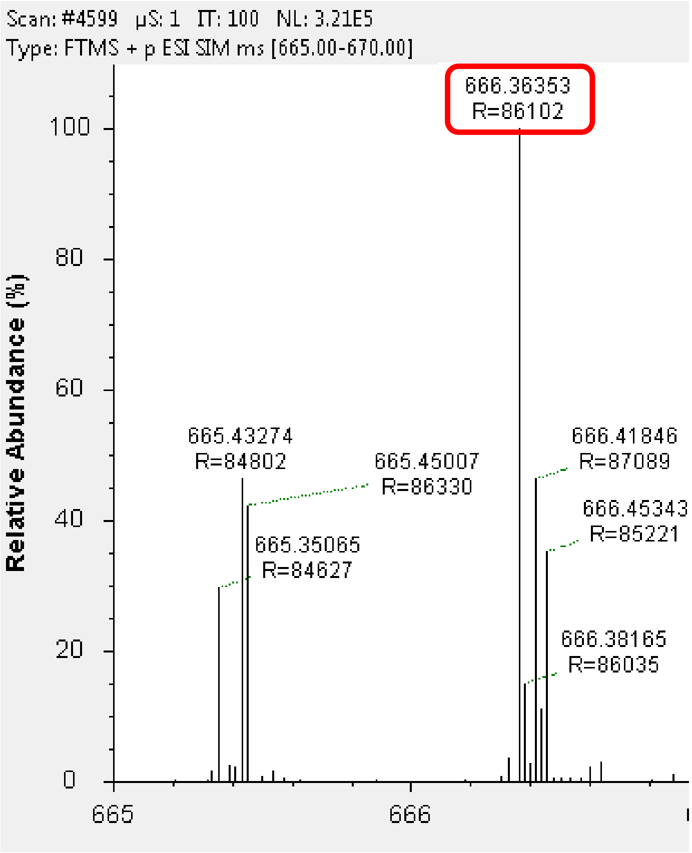

Mass-spectrum of compound 3 $[M+H]^+$ (A_4 form):

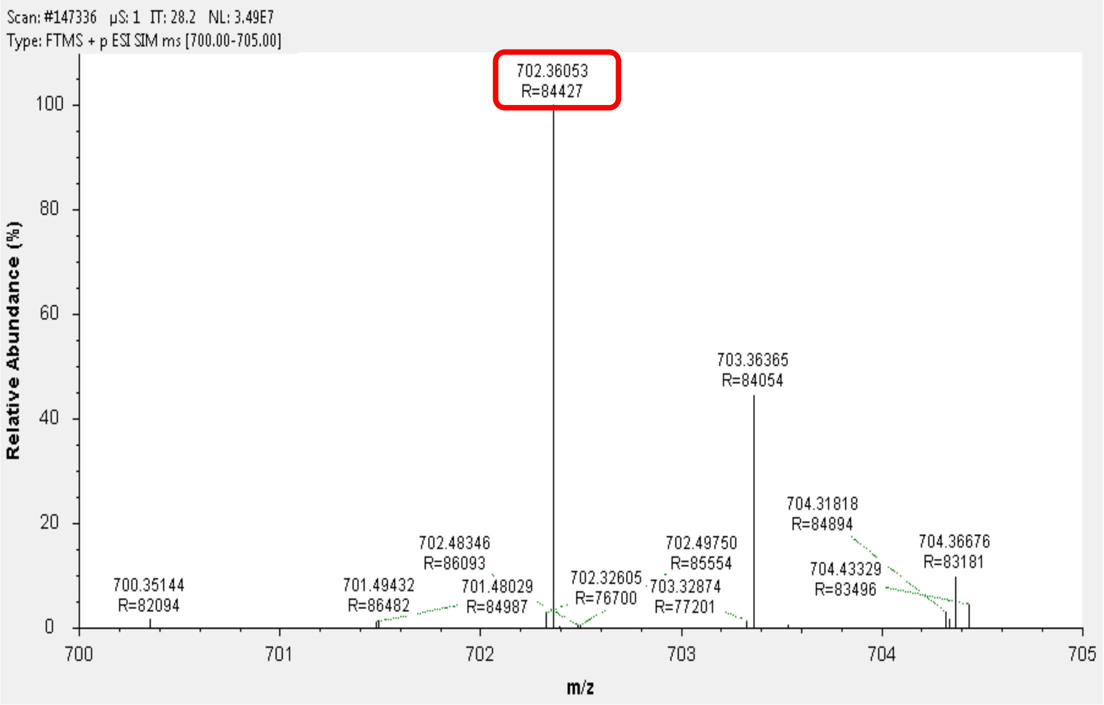

Synthesis of product M-L17 (process 2 → M-L17)

A solution of $CuSO_4 \cdot 5H_2O$ (1 mg, 0.004 mmol) in H_2O (0.5 mL) and a solution of sodium ascorbate (2 mg, 0.008 mmol) in H_2O (0.5 mL) were sequentially added to a mixture of the intermediate **2** (30 mg, 0.044 mmol) and [2-(2-azidoethoxy)ethoxy]acetic acid cyclohexylammonium salt (14 mg, 0.048 mmol) in THF (1 mL). The resulting reaction mixture was stirred at ambient temperature and its progress was controlled by HPLC (for the conditions, see above; t_R(**M-L17**$_{A3}$)=10.388 min, t_R(**M-L17**$_{A4}$)=11.043 min). DCM (10 mL) was added to the reaction mixture and the resulting mixture was washed with brine (3 × 1 mL), dried over anh. Na_2SO_4, filtered and evaporated under reduced pressure. The residue was chromatographed on silica gel (0.5% $CHCl_3$/MeOH) and yielded product **M-L17** as an oily material. The latter was dissolved in freshly distilled dioxane and lyophilized for 8 hours at 0.01 Torr to yield product **M-L17** (42 mg, 73%) in a form of white amorphous foamy material [mixture of milbemycins A_3/A_4 ~ 1/2].

Analytical data for compound M-L17 [a mixture of A_3/A_4 ~ 1/2]

^{1}H NMR ($CDCl_3$, 300 MHz): 7.91-7.77, 7.76-7.65 (2 bs, 2H), 5.84-5.69 (m, 2H), 5.59-5.47 (m, 2H), 5.43-5.32 (m, 1.5H), 5.26-5.13 (m, 1.5H), 5.04-4.92 (m, 1.5H), 4.67-4.36 (m, 7.5H), 3.98 (d, 1.5H, *J* = 5.3 Hz), 3.87-3.66 (m, 7H), 3.63-3.39 (m, 7H), 3.32-3.18 (m, 1.5H), 3.06 (t, 1.5H, *J* = 8.5 Hz), 2.85-2.65 (m, 2.5H), 2.60-2.34 (m, 4H), 2.30-2.04 (m, 4.5), 1.93-1.79 (m, 3H), 1.72-1.43 (m, 12.5H), 1.38-1.20 (m, 5.5H), 1.12 (d, 1.5H, *J* = 9.3 Hz), 0.99-0.80 (m, 11H).
^{13}C NMR ($CDCl_3$, 75.5 MHz): 176.28, 172.98, 172.64, 171.83, 145.21, 142.71, 139.12, 137.06, 132.62, 123.51 (2C), 121.30, 120.93, 120.46, 97.57 (A_3: spirocyclic C) , 97.40 (A_4: spirocyclic C), 80.33, 77.71, 75.93, 71.26 (A_3: CH), 70.80, 70.43 (A_4: CH), 70.21, 69.31 (A_4: CH_2), 69.12 (A_3: CH_2), 68.94, 68.15, 67.46, 67.09, 49.92, 48.57, 45.83, 41.43 (A_4: CH_2), 41.27 (A_3: CH_2), 38.74, 36.64, 36.44, 35.88 (A_4: CH), 35.64, 34.87 (A_4: CH_2), 34.67 (A_3: CH_2), 34.24 (A_3: CH), 30.41 (A_3: CH), 30.37 (A_4: CH_2), 29.69 (A_3: CH_2), 29.19, 27.89 (A_4: CH_2), 27.74 (A_3: CH_2), 25.69(A_4: CH_2 from Et), 22.42, 19.35, 17.89 (A_3: CH_3), 17.78 (A_4: CH_3), 15.57, 10.12 (A_4: CH_3 from Et).
HRMS (ESI): Calcd for A_3 form $[C_{44}H_{62}N_4O_{13}+H]^+$ 855.4386; found: 855.4371
HRMS (ESI): Calcd for A_4 form $[C_{45}H_{64}N_4O_{13}+H]^+$ 869.4543; found: 869.4533

^{1}H-NMR spectrum (300 MHz, $CDCl_3$) of compound M-L17:

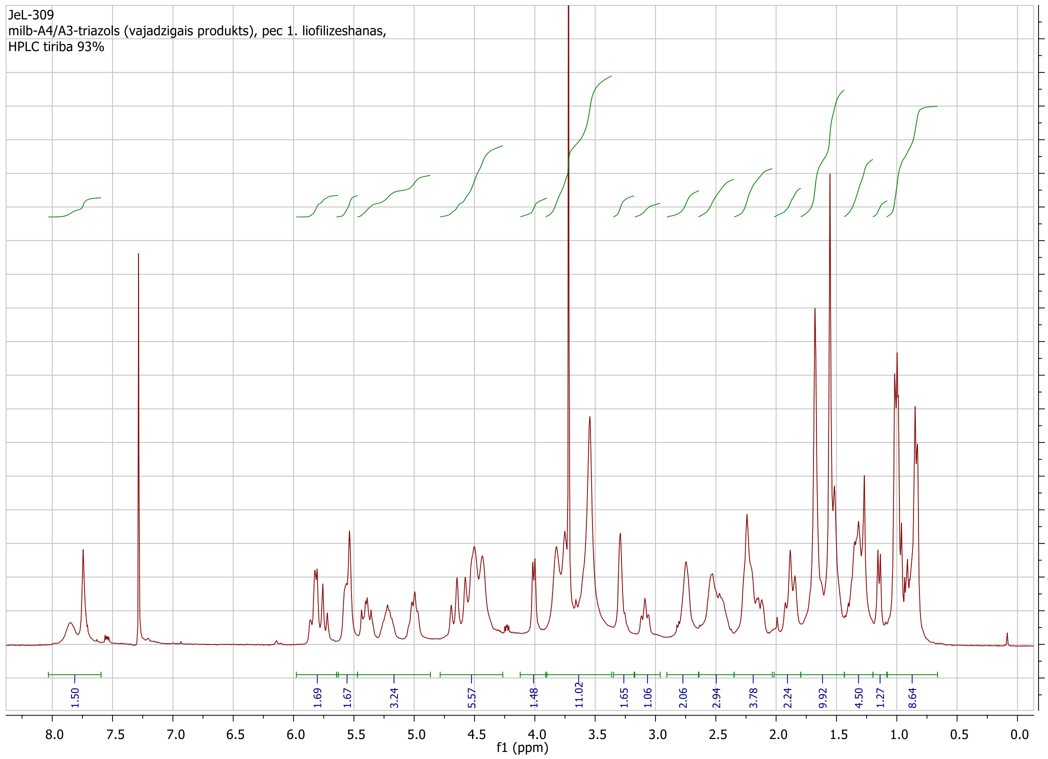

^{13}C-NMR spectrum (75.5 MHz, $CDCl_3$) of compound M-L17:

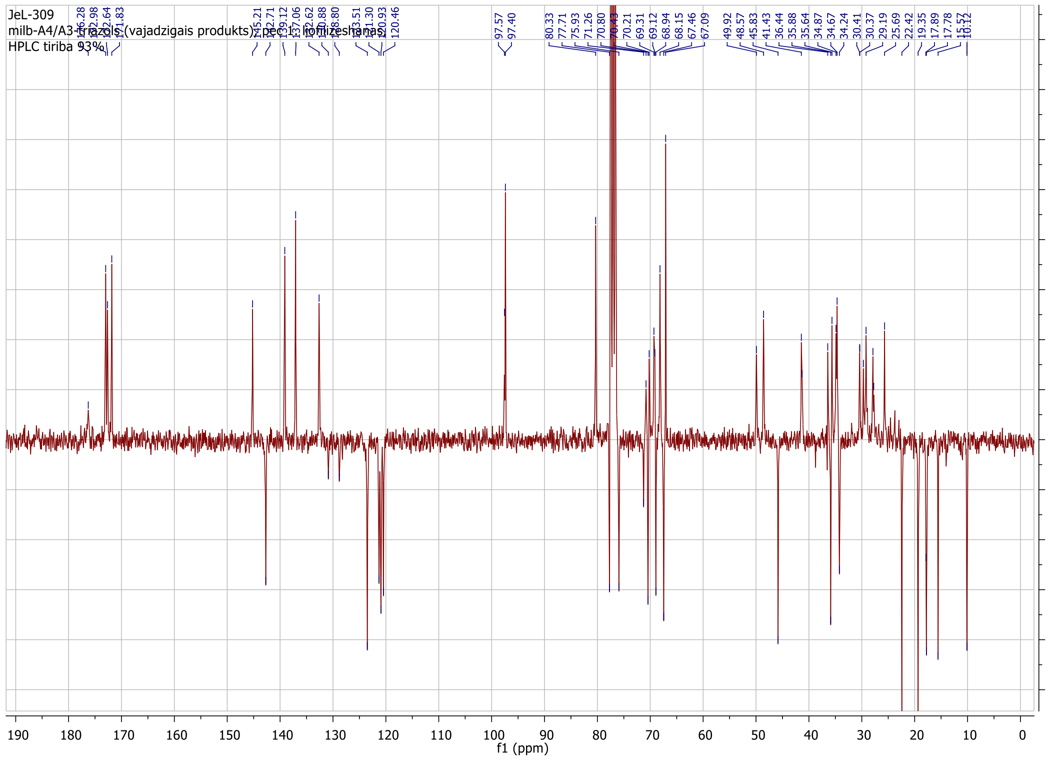

Mass-spectrum of compound M-L17 $[M+H]^+$ (A_3 form):

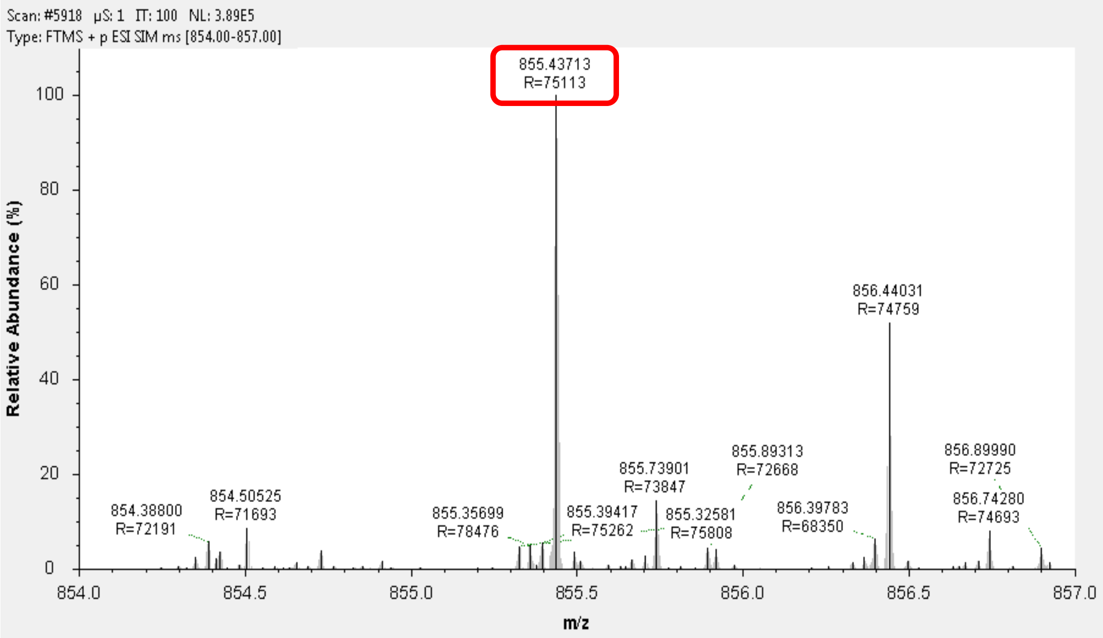

Mass-spectrum of compound M-L17 $[M+H]^+$ (A_4 form):

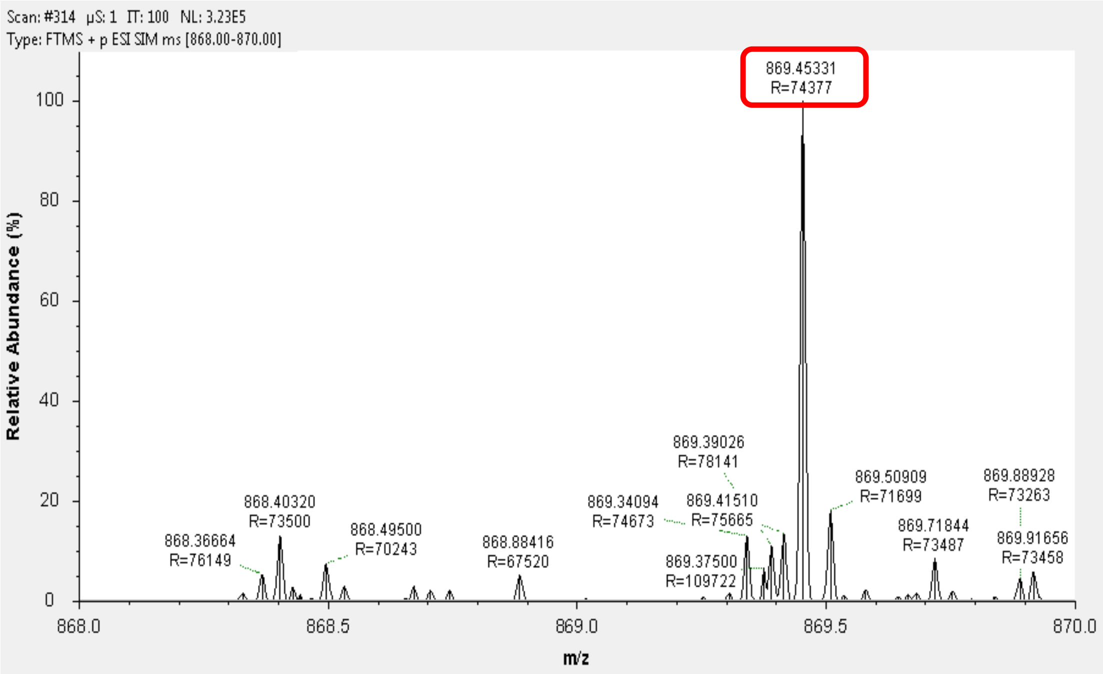

Supplement: Supplementary file 1 [file antibiotics-06-00018-s001.zip › Suppl_file1_synth_milbemycin_haptens_revised_6Aug2017_CLEAN.pdf]
