# Supplementary material for: Synthesis and Immunological Evaluation of Virus-Like Particle-Milbemycin A3/A4 Conjugates
Source: Antibiotics (Basel). 2017 Sep 11;6(3):18. doi: 10.3390/antibiotics6030018 (PMC5617982; doi:10.3390/antibiotics6030018)
Supplement: Supplementary file 1 [file antibiotics-06-00018-s001.zip › Suppl_file2_synth_M4-BSA_conjugate.pdf]

ELECTRONIC SUPPLEMENTARY MATERIAL

Synthesis and immunological evaluation of virus-like particle-milbemycin A_3/A_4 conjugates

Andris Zeltins • Māris Turks • Dace Skrastina • Jevgeņija Luginina • Ieva Kalnciema • Ina Balke • Ērika Bizdena • Vitalijs Skrivelis

Part II. Synthesis of milbemycin-M-L4-BSA conjugate

For synthesis of covering antigen M-L4-BSA, milbemycin hemisuccinate **M-L4** (6.5 mg) was dissolved in dimethylformamide (DMF;100 µl) and added to a solid mixture of EDC (1-ethyl-3- [3-dimethyl-aminopropyl] carbodiimide; 2.3 mg) and sulfo-NHS (N-hydroxysulfo-succinimide; 2.6 mg). The resulting reaction mixture was shaken for 4 h at room temperature. Then the excess of EDC was neutralized with mercaptoethanol (1.4 µl).

The reaction mixture from previous step (100 µl) containing freshly prepared sulfo-NHS activated ester was slowly added to bovine serum albumin solution (1 ml; BSA, Serva, Heidelberg, Germany; 10 mg/ml in 200 mM sodium borate, pH 8.0) and incubated for 16 h at 4 °C on a rotator mixer. The reaction was stopped by adding Tris·HCl (20 µl; 1M, pH 7.0, 1M). Visual inspection of the sample after reaction revealed a slight turbidity. Then, the M-L4-BSA conjugate was dialyzed against 200 volumes of 1xPBS buffer at 4 °C for 16 h, clarified by centrifugation (16,000 g, 10 min), and analysed by UV-spectroscopy and mass spectrometry. (Fig. SII-1).
As can be seen in Fig. SII-1, the UV spectrum of BSA was considerably changed after coupling of **M-L4**. Characteristic for milbemycins absorbance peak at 245 nm was observed after coupling of milbemycin hemisuccinate **M-L4**. Moreover, mass spectrometry analysis demonstrated that all BSA molecules were modified: no molecules with molecular weight of free BSA (66.5 kDa) were detected. Predominant molecular weight of the reaction product was 78.5 kDa (Fig. SII-1, B) corresponding to a molecular weight of BSA with approximately 17 coupled **M-L4** residues.

The M-L4-BSA conjugate was used as covering antigen in ELISA tests and as a control antigen in immunization experiments.

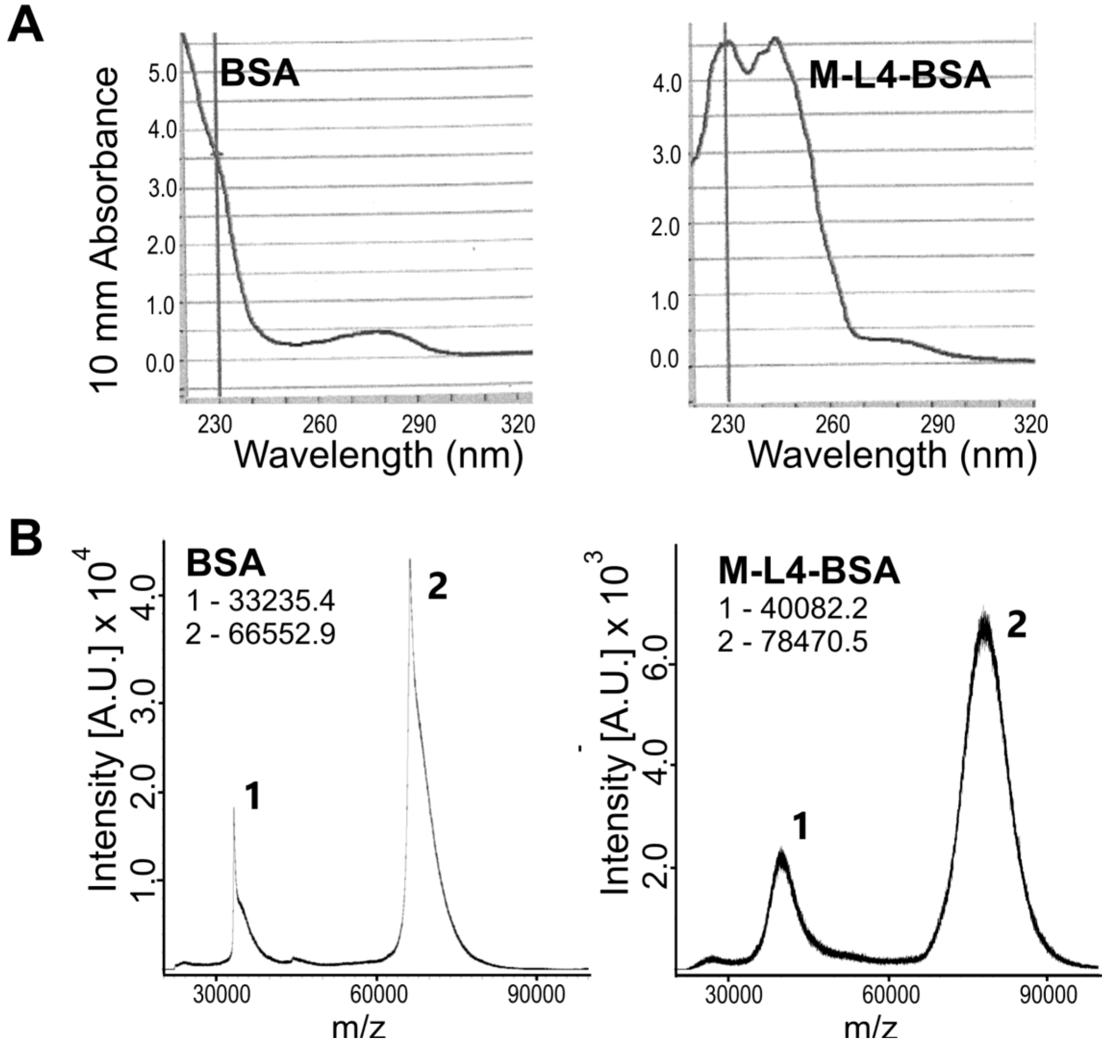

Fig. SII-1. Comparison of UV and mass spectra of unmodified bovine serum albumin (BSA) and milbemycin-M-L4-BSA conjugate (M4-BSA). A - UV spectra of BSA and M-L4-BSA derivative. The UV spectrum of protein solution (1 mg/ml) was recorded using a Nanodrop ND-1000 spectrophotometer (NanoDrop Technologies, Wilmington, USA). B – Mass spectrometric analysis. BSA samples (1 mg/ml) were diluted with a 3-hydroxypicolinic acid matrix solution and were spotted onto an MTP AnchorChip 400/384TF. Matrix-assisted laser desorption/ionization (MALDI)-TOF MS analysis was carried out on an Autoflex MS (Bruker Daltonik, Bremen, Germany). The protein molecular mass calibration standard II (22.3–66.5 kDa; Bruker Daltonik) was used for mass determination.
