# Supplementary material for: Synthesis and Immunological Evaluation of Virus-Like Particle-Milbemycin A3/A4 Conjugates
Source: Antibiotics (Basel). 2017 Sep 11;6(3):18. doi: 10.3390/antibiotics6030018 (PMC5617982; doi:10.3390/antibiotics6030018)
Supplement: Supplementary file 1 [file antibiotics-06-00018-s001.zip › Suppl_file3_dot-blots.pdf]

ELECTRONIC SUPPLEMENTARY MATERIAL

Synthesis and immunological evaluation of virus-like particle-milbemycin A_3/A_4 conjugates

Andris Zeltins • Māris Turks • Dace Skrastina • Jevgeņija Luginina • Ieva Kalnciema • Ina Balke • Ērika Bizdena • Vitalijs Skrivelis

Part III. Dot-blot tests

To evaluate the effectiveness of antibodies raised against different milbemycin-containing antigens, dot-blot assay was used. Milbemycin or M-L4-BSA covering antigen solutions (10 µl, at corresponding dilution) were spotted on unsupported nitrocellulose transfer membrane (Applichem, Darmstadt, Germany) and dried at room temperature (RT). Then, the membranes were blocked in a PBS solution containing 1% non-fat milk powder and 0.3% bovine serum albumin and incubated overnight at 4°C in corresponding antibody solution (diluted 1:100 in PBS) obtained from mice that were immunized with different milbemycin-containing antigens. The membranes were washed three times with PBS and further incubated at RT for 3 h with horseradish peroxidase-conjugated anti-mouse IgG (Sigma, Saint Louis, USA) that was diluted 1:1,000 in PBS. The signal spots were developed by incubating the membranes in TBS buffer supplemented with peroxidase substrates (0.002% o-dianisidine and 0.03% hydrogen peroxide). The results are summarized in Fig. SIII-1.

As seen in the Figure, sera from unimmunized mice did not recognized the milbemycin and M-L4-BSA conjugate, whereas sera from M-L4-BSA-immunized mice reacted with the same antigen M-L4-BSA at all concentrations tested. Sera obtained after immunization with PVY-derived carriers did not bind to unmodified milbemycin spots. The treatment of blots with Anti-M-L4-PVY polyclonal antibodies resulted in detectable signals only at high concentrations of M-L4-BSA, whereas Anti-M-L17-PVY were not able to bind the M-L4-BSA covering antigen. These results suggest, that obtained polyclonal sera contain very low amounts of milbemycin-specific antibodies and are not suitable for antibody-based milbemycin dot-blot tests.

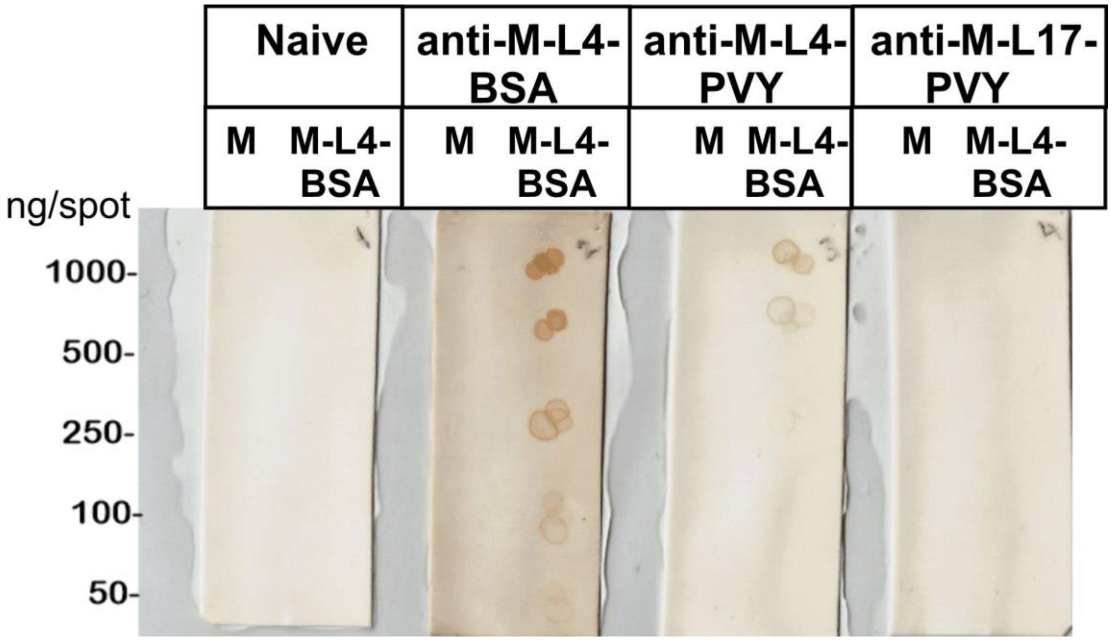

Fig. SIII-1. Dot-blot sensitivity test of obtained antibodies. Unmodified milbemycin (M) or M-L4-BSA covering antigen (M-L4-BSA) were spotted on nitrocellulose membranes (50 – 1000 ng/spot). Sera from unimmunized mice (Naive) or immunized mice (anti-M-L4-BSA; anti-M-L4-PVY and anti-M-L17-PVY) were diluted (1:100) and used in dot-blot test as described in the text.
